# Determinants of antipsychotic prescription in women detainees admitted to an acute forensic psychiatric unit

**DOI:** 10.3389/fpsyt.2026.1776130

**Published:** 2026-05-19

**Authors:** Isabella D’Orta, Agathe Nobis, Kerstin Weber, François R. Herrmann, Panteleimon Giannakopoulos

**Affiliations:** 1 Division of Institutional Measures, Department of Psychiatry, Geneva University Hospitals, Geneva, Switzerland; 2 Institute of Global Health, University of Geneva, Geneva, Switzerland; 3 Center for the Interdisciplinary Study of Gerontology and Vulnerability, University of Geneva, Geneva, Switzerland; 4 Department of Psychiatry, University of Geneva, Geneva, Switzerland; 5 Department of Rehabilitation and Geriatrics, Geneva University Hospitals and University of Geneva, Geneva, Switzerland; 6 Health Office, Republic of Geneva, Geneva, Switzerland

**Keywords:** antipsychotics, court-ordered treatements, gender, off-label, mental health of detainees, prison health

## Abstract

**Background:**

Women represent a small proportion of the global prison population but carry a disproportionate burden of mental illness. Evidence indicates high rates of psychotropic medication use among women in prison, often independently of the clinical diagnosis. Antipsychotics, particularly second-generation (SGAs) are widely prescribed in forensic settings. Data focusing on the prescription of these agents to women detainees are still scarce.

**Objectives:**

This study aimed to investigate whether socio-demographic, clinical and forensic characteristics determine SGA prescription to women admitted in an acute forensic psychiatric ward located in prison.

**Methods:**

We conducted a retrospective study of 166 women admitted between 2014 and 2023 to the sole acute forensic psychiatric unit for detainees in French-speaking Switzerland. Among them, 128 cases received second generation antipsychotic medication during their hospital stay. Sociodemographic data included age, nationality, educational attainment and primary spoken language. Criminological variables included types of offense and detention. Clinical variables included psychiatric outpatient and inpatient history prior to conviction, total number of stays during the study period, main diagnosis, presence of substance use disorder and personality disorders. Psychotropic prescriptions were analyzed with conversion of antipsychotic doses into chlorpromazine equivalents. Regression analyses, including LASSO models were used to identify variables associated with antipsychotic dosage.

**Results:**

Psychotropic use was very high with more than two-thirds receiving two or more psychotropic agents. SGAs were prescribed in 77.1% of cases (128 out of 166 cases), while psychotic disorders were diagnosed in only 28.3% indicating frequent off-label use. Regression analyses showed that higher antipsychotic doses were associated with previous psychiatric history, more inpatient stays and court-ordered treatments. Cluster A personality disorders were associated with lower antipsychotic doses.

**Conclusions:**

Our findings reveal an extremely high rate of psychotropic use with very frequent off-label prescription for antipsychotics in acute forensic psychiatry wards. Moreover, they show that clinical variables and not demographic and criminological factors determine the use of antipsychotics in this setting.

## Introduction

Women constitute a minority of the general prison population worldwide, with estimates ranging from 2% to 9% of a country’s prison population. This corresponds to over 740,000 women and girls held in prisons worldwide, either on remand or sentenced detainees (1–3). There is evidence that women in prison often come from disadvantaged backgrounds and are more likely than men to experience poor mental health (2). These mental health challenges are often linked to histories of domestic violence, physical abuse, and sexual trauma (2, 3). Evidence suggests that mental health problems are increasing in prison populations, especially in women (4, 5). Until the beginning of the new century, forensic studies on women were based on the tacit assumption that the psychopathology observed in men could be applied to women, without taking gender sensitivity into account (6).

Several lines of evidence support the existence of significant gender-related differences in psychiatric morbidity in prison. Incarcerated women experience a higher burden of mental illness compared to men, use more psychotropic agents and are more exposed to suicide and overdose deaths following release (7–9). The overall increase in psychiatric morbidity in prisons in recent decades is thought to be even more pronounced among women with high rates of major depression, anxiety and substance use disorders (SUD) (4, 10–14). Women referred to forensic outpatient services displayed more often psychotic disorders with comorbid SUD (15). Those with personality disorders need more frequently acute forensic care but are significantly less frequently sentenced to court-ordered treatments than men (13, 16, 17).

Psychotropic prescription in prisons is a complex and controversial area. Prescribers must balance individual clinical needs against security risks. Overall, psychotropic agents appear to be prescribed more frequently in prisons than in the community and across a broader range of clinical indications, most often off-label for low mood and personality disorders (18). This phenomenon is more pronounced for women with almost half of them receiving one or more psychotropics in prison compared to less than 20% for men (8, 19). Compared with the general population, age-adjusted prescribing prevalence could be six times higher among women (19).

Antipsychotics were the most frequently used psychotropic medication class with more than 80% of patients receiving at least one typical or atypical molecule at admission in acute forensic psychiatry care (20). Although in prisons these rates are lower, off-label prescriptions are very frequent with less than one-third of all antipsychotic prescriptions being made for psychotic disorders (21–23). Women detainees received higher doses of oral treatments and more frequent prescription of second-generation depot antipsychotics (8, 9, 24, 25). The prescription of antipsychotics is associated with lower rates of criminal offenses among women (26, 27). Yet, the increased use of antipsychotics in women detainees is also associated with disproportionately higher rates of weight gain and metabolic syndromes (28). The prescription of antipsychotics in women detainees may depend on numerous factors including demographic (age, education, nationality, language), criminological (type of offenses, type of detention) and clinical (previous inpatient stays, history of outpatient and inpatient psychiatric care prior to incarceration, presence of substance use disorders, and types of personality disorders) parameters. Despite these concerns, the determinants of antipsychotic prescribing in incarcerated women remain poorly understood.

In this study, we explore the determinants of the prescription of second-generation antipsychotics (SGAs) in a total sample of 166 women hospitalized in the sole Geneva forensic unit for acute care during a 10-year period. Our main hypothesis is that previous psychiatric history and number of inpatient stays rather than the sole diagnosis of psychotic disorders would determine the prescription of SGAs in women admitted in this setting.

## Materials and methods

### Study sample and data collection

We retrospectively examined the psychiatric records corresponding to all admissions of women during a 10-year period (2014-2023) in the UHPP (Unité hospitalière de psychiatrie penitentiaire), a unique ward of 15 beds specially designed for acute psychiatric care of prison detainees from the French speaking counties in Switzerland. This unit is located in a medium-security hospital (Curabilis) that is also in charge of the COT (Court Ordered Treatment) for French -speaking offenders in Switzerland. Women were admitted to this ward under three possible statuses: pre-trail detention (PTD), sentence execution (SE) and COT. The total mean number of admissions per year for the period of reference was of 261. Patients are admitted to the UHPP in the presence of acute symptoms associated with self or others-threatening behavior and need for urgent psychiatric care. The sample of reference (women with and without antipsychotic medication) included 166 women (mean age: 36.7 ± 11.2, age range: 22-59).

The study focuses exclusively on women. No transgender female patients were admitted during this period; therefore, the sample included exclusively cisgender women. The two inclusion criteria were being female and having received an antipsychotic prescription during the stay in the unit. Among the 166 women (70 PTD, 61 SE and 35 COT cases (art 59, 60 Swiss Criminal Code)) admitted during the study period, 128 received second generation antipsychotic treatment. Every patient was assigned an identification number that was derived from the name and birth date and subsequently encrypted. Sociodemographic data included age, nationality, educational attainment and primary spoken language (French or other). Criminological variables included type of offense (according to the Swiss Penal Code; https://www.fedlex.admin.ch/eli/cc/54/757_781_799/fr) and type of detention. Clinical variables included psychiatric outpatient and inpatient history prior to conviction, main ICD-10 diagnosis, presence of substance use disorder (SUD), types of personality disorders and total number of stays during the study period. Given the limited number of cases with inpatient stays prior to conviction, they were grouped with cases with outpatient psychiatric history. Psychiatric diagnoses were extracted as documented in the psychiatric reports. As part of the routine procedure, they were made at admission by two independent, board-certified psychiatrists, only concordant diagnoses were included in the present study.

Prescriptions for all psychotropic agents were recorded, including the number of molecules. Daily antipsychotic doses were converted into chlorpromazine (CPZ) equivalents to allow comparisons across drugs. CPZ equivalents are intended to reflect relative antipsychotic potency across agents and to uniform the dosage for comparison.

The Defined Daily Dose (DDD) is the international unit approved by the World Health Organization for drug use studies (29). It represents the assumed average maintenance dose daily for a drug used for its main indication in adults (30). Unlike CPZ equivalents, which are based on relative pharmacodynamic potency, the DDD is a drug utilization metric designed for pharmacoepidemiologic standardization rather than direct potency comparison.

The DDD system has shown a high level of concordance with chlorpromazine equivalent doses and is considered a reliable tool for standardizing antipsychotic dosing in drug utilization studies (31, 32). For example, the DDDs of chlorpromazine are 300 mg/day, of haloperidol 8 mg/day, risperidone 5 mg/day and olanzapine 10 mg/day (30). Therefore, 1 mg/day of haloperidol, risperidone, and olanzapine are 37.5 (= 300/8), 60 (= 300/5), and 30 (= 300/10) mg/day of CPZ equivalent doses. The combination of oral and/or intramuscular antipsychotic administration was analyzed and classified into three categories: oral only, intramuscular only, both routes.

In the present study, DDD values were used to estimate CPZ equivalents in order to ensure consistent cross-drug standardization. We acknowledge that DDD-based CPZ equivalence represents an approximation of dose equivalence rather than a direct measure of pharmacodynamic potency.

### Statistical analysis

Fisher exact, unpaired Student t and Mann-Whitney u tests were used to compare sociodemographic criminological and clinical variables as a function of single versus multiple users (referring to single versus multiple admissions during the study period). The prescription of antipsychotics was examined both qualitatively and using chlorpromazine equivalents based on the mean dose of antipsychotics during each hospital stay. Age was treated as quantitative variable. Nationality (EU, extra EU, Swiss), education (drop-out, obligatory, high school and university, apprenticeship), type of offenses (arson, road traffic law, illegal immigration, drug related offense (drug trafficking and minor drug possession), violation of property, minor theft, physical violence, white collar-fines),and number of stays (1, 2-10, >10) were treated as ordinal variables. Previous outpatient and inpatient care, fluent French and SUD were treated as binary variables. Psychiatric diagnoses included adjustment disorders, bipolar and depressive disorders (ICD-10 codes F32-33), personality disorders (F60), and psychosis (ICD-10 codes F20-F29). The distribution of personality disorders included the distinction between the most frequently occurred borderline, narcissistic and histrionic types (n = 65), other types of personality disorders (n = 14) and none. The significance level was set at P < 0.05 but was corrected to P < 0.00625 for multiple testing by using the Benjamini-Hochberg method (Green and Diggle, 2007). LASSO (least absolute shrinkage and selection operator) linear regression models were used as a variable selection method to detect independent variables associated with antipsychotic chlorpromazine equivalents. The significance level was set at P < 0.05. All analyses were performed with Stata release 18.5 (College Station, Texas, USA).

## Results

Compared to women with multiple admissions, women with single referral during the period of observation were more frequently first time incarcerated and had less often a previous psychiatric history. Of note, however, more than 80% of women detainees had outpatient psychiatric follow-up prior to incarceration. These two differences persisted after correction for multiple comparisons. Of importance, neither criminological nor clinical factors differed between the two groups. Most importantly, there were no significant group differences in both the number of psychotropic agents, and frequency of prescription of antipsychotics. More than 69% of women received 2 to 3 psychotropic agents during their hospital stays. Second generation antipsychotics (SGA) were prescribed in 77.1% of cases, just after anxiolytics (77.7%). Prescription of antidepressants was made in 40.4% of cases. Contrasting with the frequency of prescription of SGA and AD, psychotic disorders were present in 28.3% of cases whereas depressive and anxious disorders concerned 21.1% of women detainees (**Table 1**).

**Table 1:** Demographic, clinical and juridical characteristics of participants (including women with and without antipsychotic treatment) according to the number of admissions (single versus multiple) during the period of reference.

	Single admission	Multiple admissions	Total	P	sig	sig BH
N	107(64.5%)	59 (35.5%)	166(100.0%)			
Age	38.257 (10.476)	38.316 (11.511)	38.278 (10.820)	0.6769		
Length of stay	21.701 (21.971)	19.475 (25.137)	20.910 (23.095)	0.2258		
Language- French	88 (82.2%)	54 (91.5%)	142 (85.5%)	0.1136		
Marital status						
Single	51 (47.7%)	37 (62.7%)	88 (53.0%)	0.0690		
Separated-divorced-widowed	38 (35.5%)	11 (18.6%)	49 (29.5%)			
Partnership	18 (16.8%)	11 (18.6%)	29 (17.5%)			
Nationality						
Adopted (extra-EU born swiss citizens)	4 (3.7%)	3 (5.1%)	7 (4.2%)	0.7545		
Europe	41 (38.3%)	19 (32.2%)	60 (36.1%)			
Extra-EU	33 (30.8%)	17 (28.8%)	50 (30.1%)			
Swiss	29 (27.1%)	20 (33.9%)	49 (29.5%)			
Measure	30 (28.0%)	24 (40.7%)	54 (32.5%)	0.1195		
Type of detention						
Pre-trial	45 (42.1%)	25 (42.4%)	70 (42.2%)	0.8760		
Sentenced	41 (38.3%)	20 (33.9%)	61 (36.7%)			
Court-ordered treatments	20 (18.7%)	13 (22.0%)	33 (19.9%)			
End of detention	1 (0.9%)	1 (1.7%)	2 (1.2%)			
First-time detained	77 (79.4%)	23 (39.0%)	100 (64.1%)	<0.0010	*	*
No	20 (20.6%)	36 (61.0%)	56 (35.9%)			
Type of offenses						
Arson	6 (5.7%)	2 (3.4%)	8 (4.9%)	0.2045		
Road traffic law	2 (1.9%)	0 (0.0%)	2 (1.2%)			
Illegal immigration	2 (1.9%)	2 (3.4%)	4 (2.5%)			
Drug related offense (drug trafficking and minor drug possession)	17 (16.2%)	10 (17.2%)	27 (16.6%)			
Violation of property	18 (17.1%)	15 (25.9%)	33 (20.2%)			
Minor theft	20 (19.0%)	12 (20.7%)	32 (19.6%)			
Physical violence	30 (28.6%)	17 (29.3%)	47 (28.8%)			
White collar-fines	10 (9.5%)	0 (0.0%)	10 (6.1%)			
Psychiatric history	84 (78.5%)	55 (93.2%)	139 (83.7%)	0.0153	*	
Compulsory admission	49 (45.8%)	26 (44.1%)	75 (45.2%)	0.8715		
Diagnosis						
Adjustment disorder/stress disorder	2 (1.9%)	0 (0.0%)	2 (1.2%)	0.1054		
Mood disorder	27 (25.2%)	6 (10.2%)	33 (19.9%)			
Personality disorder	19 (17.8%)	10 (16.9%)	29 (17.5%)			
Psychotic disorder	27 (25.2%)	20 (33.9%)	47 (28.3%)			
Other	32 (29.9%)	23 (39.0%)	55 (33.1%)			
Substance use disorder	46 (43.0%)	33 (55.9%)	79 (47.6%)	0.1438		
Personality						
None	62 (57.9%)	25 (42.4%)	87 (52.4%)	0.1925		
Not specified	7 (6.5%)	5 (8.5%)	12 (7.2%)			
Borderline	34 (31.8%)	26 (44.1%)	60 (36.1%)			
Cluster A	2 (1.9%)	0 (0.0%)	2 (1.2%)			
Other Cluster B	2 (1.9%)	3 (5.1%)	5 (3.0%)			

Benjamini-Hochberg adjusted p-threshold = 0.004.

The LASSO linear regression models identified four variables associated with antipsychotic chlorpromazine equivalents in our women sample: number of hospital stays, previous psychiatric history, court-ordered treatments and cluster A personality disorders. The three first variables were positively related to chlorpromazine equivalents whereas the presence of cluster A personality disorders is associated with decreased levels of antipsychotic prescription. There was no interaction between court ordered treatments and past psychiatric history. In multivariable models, the combination of the four variables explained 13.3% of the variance in the dependent variable (**Table 2**).

**Table 2:** Number and type of prescribed drugs.

	Users			P	sig	sig BH
	Single	Multiple	Total			
**N**	107 (64.5%)	59 (35.5%)	166 (100.0%)			
Number of prescribed drugs						
0	6 (5.6%)	2 (3.4%)	8 (4.8%)	0.1085		
1	14 (13.1%)	9 (15.3%)	23 (13.9%)			
2	51 (47.7%)	18 (30.5%)	69 (41.6%)			
3	27 (25.2%)	19 (32.2%)	46 (27.7%)			
4	5 (4.7%)	9 (15.3%)	14 (8.4%)			
5	4 (3.7%)	2 (3.4%)	6 (3.6%)			
Type of prescribed drugs						
Antipsychotics	81 (75.7%)	47 (79.7%)	128 (77.1%)	0.6999		
Antidepressants	41 (38.3%)	26 (44.1%)	67 (40.4%)	0.5107		
Anxiolytics	79 (73.8%)	50 (84.7%)	129 (77.7%)	0.1219		
Mood stabilizers	15 (14.0%)	10 (16.9%)	25 (15.1%)	0.6535		
Antipsychotics IM	2 (1.9%)	3 (5.1%)	5 (3.0%)	0.3484		
Antipsychotics Oral	2 (1.9%)	2 (3.4%)	4 (2.4%)	0.6161		
Antipsychotics IM+ Oral	5 (4.7%)	6 (10.2%)	11 (6.6%)	0.2007		
Methadone	12 (11.2%)	4 (6.8%)	16 (9.6%)	0.4215		

Benjamini-Hochberg adjusted p-threshold = 0.004.

Cluster A personality disorders are a group of diagnoses characterized by odd, eccentric or unusual patterns of thinking and behavior, and particularly interpersonal functioning. They are grouped together in the Diagnostic and Statistical Manual of Mental Disorders (DSM-5-TR) due to shared features of social detachment, suspiciousness and cognitive or perceptual abnormalities (33, 34). They can resemble, but do not reach the threshold for psychotic disorders.

Cluster A includes

Paranoid personality disorder: marked by pervasive distrust and suspiciousness of others.

Schizoid personality disorder: characterized by detachment from social relationships and restricted emotional expression.

Schizotypal personality disorder: defined by discomfort in close relationships, odd beliefs or magical thinking, and eccentric behavior.

These conditions are stable, enduring patterns that typically begin in early adulthood and reflect maladaptive personality traits affecting cognition, affectivity, and interpersonal functioning (33, 34).

Results of univariate and multiple regression models are shown in **Table 3**.

**Table 3:** Results of univariate and multiple regression models (R² = 13.3%).

	Univariate				Multiple		
	Coeff crude	95% CI	P	R^2^	Coeff adjusted	95% CI	P
Number of admissions (multiple versus single)	20.69	[11.35, 30.03]	< 0.001	10.4	17.22	[7.85, 26.58]	< 0.001
Previous psychiatric history	142.46	[20.25, 264.67]	0.023	3.8	135.31	[16.97, 253.65]	0.025
Court-ordered treatments	440.26	[115.28, 765.24]	0.008	10.1	349.30	[33.34, 665.26]	0.030
Previous psychiatric history # Court-ordered treatments					-216.05	[-542.01, 109.91]	0.192
Personality				3.3			
Cluster A	-179.95	[-340.90, -19.01]	0.029		-192.93	[-338.66, -47.21]	0.010
Other Cluster B	13.23	[-78.41, 104.86]	0.776		-68.31	[-155.55, 18.92]	0.124

## Discussion

Our data reveal the patterns of prescription of psychotropics medication with special focus on SGA in women detainees admitted to an acute forensic psychiatry unit. They confirm the extremely high prevalence of psychotropic use, including the very frequent off-label prescription of both antipsychotics and antidepressants. Moreover, the findings indicate that in this setting, antipsychotic use is more likely driven by clinical variables rather than by demographic or criminological factors.

From a clinical standpoint, detainees incarcerated for the first time with positive psychiatric history prior to incarceration are mainly representative of the women admitted in our ward.

In agreement with previous contributions, borderline personality disorders (BPD) were the most frequent diagnosis followed by psychosis (13, 16, 17). Of importance, outpatient psychiatric care prior to conviction was present in more than 80% of cases suggesting a pre-existing psychiatric burden in women admitted for acute psychiatric care in prison. The use of psychotropic medication was frequent and mainly concerned anxiolytics and antipsychotics and, to a lesser extent, antidepressants.

**Figure 1:**
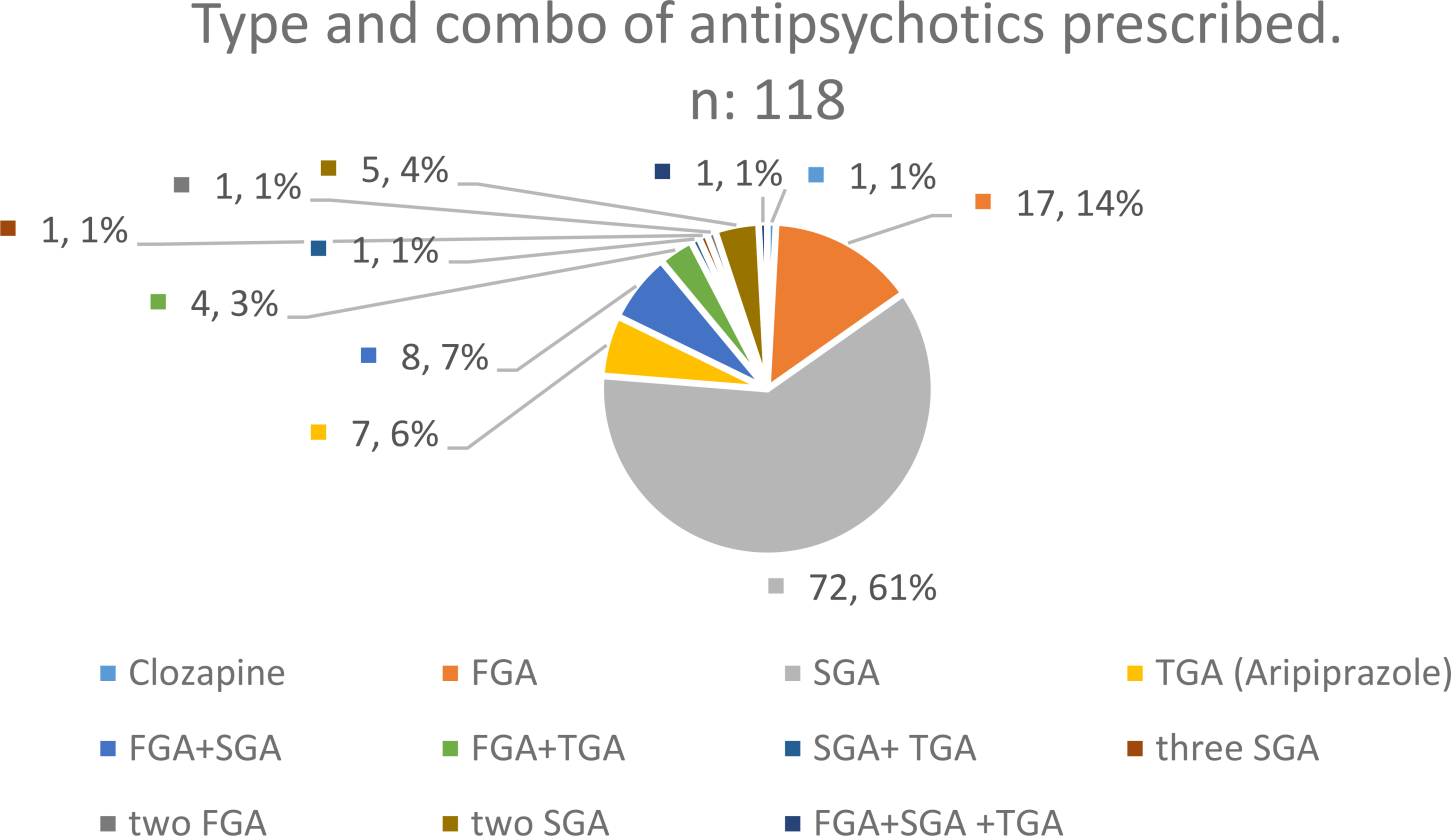
Type of antipsychotic and combo prescribed. First generation Antipsychotics (FGA): Haloperidol, Clotiapine, Flupentixolc, Levomepromazine, Zuclopenthixol. Second generation Antipsychotics (SGA): Olanzapine, Quetiapine, Risperidone, Amisulpride. Third Generation Antipsychotics (TGA): Aripiprazole, Brexpiprazole, Cariprazine.

Polypharmacy was also present with more than 69% of cases receiving 2 to 3 psychotropic drugs. In agreement with the rare previous reports in this field (8, 9, 24), off-label prescription of antipsychotics but also antidepressants was the rule in our sample. Pointing to this fact, the gap between formal diagnosis of depressive and anxious disorders and antidepressant prescription was very significant (21.1% versus 40.4%). This was even more pronounced with respect to antipsychotics (28.3% versus 77.1%). Type of antipsychotic and combo prescribed are shown in **Figure 1**.

First-time incarceration in women with a past psychiatric history is associated with the use of multiple psychotropic agents, often unrelated to the underlying diagnosis (10, 35, 36). Taken together, these descriptive data stress the increased vulnerability of women detainees admitted in acute psychiatric wards.

Given the extremely high rate of SGAs prescription in our setting, we explored the determinants of antipsychotic chlorpromazine equivalents using regression analyses. Our findings show that clinical variables beyond diagnosis, rather than demographic or criminological factors, predict antipsychotic dosage in this setting. Among them, the number of previous inpatient stays, outpatient care prior to incarceration and court-ordered treatments were positively related to this variable. As our descriptive data, these observations confirm that long-standing psychiatric burden rather than a specific diagnosis is the main predictor of antipsychotic use in women detainees.

Interestingly, the presence of cluster A personality disorders was related to the use of lower doses of antipsychotics. At first glance surprising, this finding may be interpreted as a function of the poor results and well-known resistance of these patients to antipsychotics (25, 26). From a clinical point of view, this may reflect clinicians’ awareness of the limited impact of pharmacological treatment on core personality traits and the risk of adverse effects. Lower antipsychotic doses may also reflect the attempts to manage circumscribed symptoms (e.g.: anxiety, suspiciousness, aberrant behaviors) rather than treating a full psychotic syndrome. Importantly, the dose of antipsychotics was not related to the type of detention and first-time incarceration suggesting that their prescription does not reflect the occurrence of psychological breakdown during the period of adaptation following the criminal conviction.

Strengths of the present study is admission of all cases in the same unit of acute psychiatric care in prison that decreases the variability in the admission criteria, multidimensional characterization of the sample including sociodemographic, clinical and criminological parameters, and use of multivariable models controlling for the variables that could impact on the prescription of psychotropics. Several limitations should, however, be mentioned. Clinical diagnosis was carried out by two independent clinicians blinded to the aim of the study. Standardized diagnostic questionnaires were not used in order to be close to a real-life situation. The assessment of previous inpatient stays outside the Geneva County was based on self-report and could be biased. Although the difference in time spent in prison may indirectly influence the prescription of antipsychotics, the absence of significant differences in psychotropic prescription between single and multiple users did not support this hypothesis.

Our observations concern a specialized unit of forensic psychiatry located in prison and not in a psychiatric hospital. Future studies in larger samples using standardized assessment of clinical diagnosis, detailed assessment of previous psychiatric history, and inclusion of forensic psychiatry units outside the prison are needed to explore the determinants of the prescription of antipsychotics in acute care forensic psychiatry settings.

## Data Availability

The raw data supporting the conclusions of this article will be made available by the authors, without undue reservation.
